# Supplementary material for: HIVprotI: an integrated web based platform for prediction and design of HIV proteins inhibitors
Source: J Cheminform. 2018 Mar 9;10:12. doi: 10.1186/s13321-018-0266-y (PMC5845081; doi:10.1186/s13321-018-0266-y)
Supplement: Supplementary file 2 — Additional file 2. Source code of HIVProtI web server. [file 13321_2018_266_MOESM2_ESM.zip › HIVprotI_Source-code/hivprotI/weka-3-6-6/doc/weka/core/mathematicalexpression/package-summary.html]

weka.core.mathematicalexpression


---


|  |  |  |
| --- | --- | --- |
| **Overview** \| **Package** \| Class \| **Tree** \| **Deprecated** \| **Index** \| **Help** \| **Weka's home** | |  |
| **PREV PACKAGE**   **NEXT PACKAGE** | **FRAMES**    **NO FRAMES**     **All Classes** |  |


---

## Package weka.core.mathematicalexpression

| **Interface Summary** | |
| --- | --- |
| **sym** | CUP generated interface containing symbol constants. |

| **Class Summary** | |
| --- | --- |
| **Parser** | CUP v0.11a beta 20060608 generated parser. |
| **Scanner** | A scanner for mathematical expressions. |

---


|  |  |  |
| --- | --- | --- |
| **Overview** \| **Package** \| Class \| **Tree** \| **Deprecated** \| **Index** \| **Help** \| **Weka's home** | |  |
| **PREV PACKAGE**   **NEXT PACKAGE** | **FRAMES**    **NO FRAMES**     **All Classes** |  |


---
